# Regeneration and repair in the healing lung

**DOI:** 10.1002/cti2.1152

**Published:** 2020-07-06

**Authors:** Andrew Lucas, Joe Yasa, Michaela Lucas

**Affiliations:** ^1^ School of Biomedical Sciences The University of Western Australia (UWA) Perth WA Australia; ^2^ Centre for Cell Therapy and Regenerative Medicine School of Medicine and Pharmacology The University of Western Australia (UWA) Perth WA Australia; ^3^ School of Medicine and Pharmacology The University of Western Australia (UWA) Perth WA Australia

**Keywords:** inflammation, lung, regeneration, repair, therapeutics

## Abstract

The lung achieves an efficient gas exchange between a complex non‐sterile atmosphere and the body via a delicate and extensive epithelial surface, with high efficiency because of elastic deformation allowing for an increase and decrease in volume during the process of breathing and because of an extensive vasculature which aids rapid gas diffusion. The lungs’ large surface area exposes the organ to a continual risk of damage from pathogens, toxins or irritants; however, lung damage can be rapidly healed via regenerative processes that restore its structure and function. In response to sustained and extensive damage, the lung is healed via a non‐regenerative process resulting in scar tissue which locally stiffens its structure, which over time leads to a serious loss of lung function and to increasing morbidities. This review discusses what is known about the factors which influence whether a lung is healed by regeneration or repair and what potential new therapeutic approaches may positively influence lung healing.

## Graphical abstract

This review discusses what is known about the detection, response and resolution of lung damage and what might influence whether a lung is healed by regeneration or repair. It also presents potential therapeutic approaches that may positively influence lung healing.

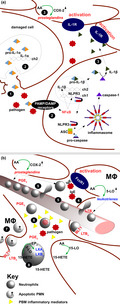

## Introduction

The oxygenation of vertebrate tissues relies on lung structures that are both delicate and vulnerable to damage. Over a human lifespan, the lungs are exposed to hundreds of millions of litres of non‐sterile air whilst maintaining their function and integrity. Despite endemic respiratory infections, lung immunity is normally sufficient for resolution without lasting structural damage. Such favorable outcomes have been significantly enhanced through the use of therapeutic antibacterial treatments. Nevertheless, sustained or more extensive lung infections can occur resulting in the accumulation of fibrotic damage which chronically affects the lungs’ flexibility and oxygen permeability.^1^ Contributing factors that can affect the severity and frequency of respiratory infections include a predisposition to develop asthma, deficiencies in immunity and/or access to appropriate vaccinations, and mutations that directly affect lung physiology such as in cystic fibrosis. Furthermore, the sustained exposure to toxins, such as a smoking habit and/or extended exposure to high levels of airborne pollutants, can directly damage lung tissue and contribute to less effective clearance of lung infections.^2^ Despite clinically identifiable risk factors, it is not possible to diagnose and intervene early enough to prevent progressive degenerative fibrotic lung diseases.

This review seeks to compare physiological lung regeneration following damage with repairs which result in scar tissue disrupting normal lung structure. We analyse the contribution of the immune response to these outcomes. Undoubtedly, the level of inflammation within the lung in the aftermath of tissue injury is one of the key factors that regulates physiological versus fibrotic repair. We discuss the latest research developments in the field, ultimately trying to understand whether we can harness this knowledge to modulate the inflammatory response in damaged lungs to enhance the lungs’ regenerative potential.

## What determines how lungs heal following damage?

The high vulnerability of the lungs to damage is associated with its structure. Firstly, the lung has a very low density of cells relative to its volume. Secondly, the functional efficiency of the lung is dependent on the organisation and composition of a range of cell types whose structural characteristics are responsible for a combination of elasticity and permeability. The most vulnerable lung cells to damage are those located at the mucosal surface. Damage to the epithelial cells stimulates rapid proliferation, differentiation and recruitment of replacement cells and can result in the regeneration of the barrier function of the tissue. However, alteration of the cellular organisation caused by repair processes that involve scarring necessarily alters critical structural characteristics, leading to poorer lung function. Factors such as the amount and cell types damaged, the disruption of barrier function and the strength and duration of the local immune response, can determine whether a damaged lung regenerates, is repaired or fails to repair leading to chronic lung disease.

## An early response matters

The disruption of tissue homeostasis caused by damage rapidly changes the local microenvironment adjacent to the damaged lung. The wound environment becomes positive for molecules derived from pathogens which contaminate it and from the intracellular environment of dying cells. The survival advantage given by the rapid detection of tissue damage has driven the evolution of cellular receptors that recognise conserved structural elements frequently found in pathogens and on intracellular cell components that are only released following damage. Such receptors are termed pattern recognition receptors (PRR) and include the Toll‐like receptors and the retinoic acid‐inducible gene I (RIG‐1)‐like receptors. Molecules derived from pathogens and recognised by specific PRR are called pathogen‐associated molecular patterns (PAMPs), whilst ligands that derived from damaged host cells are known as danger‐associated molecular patterns (DAMPs).^3^

**Figure 1:**
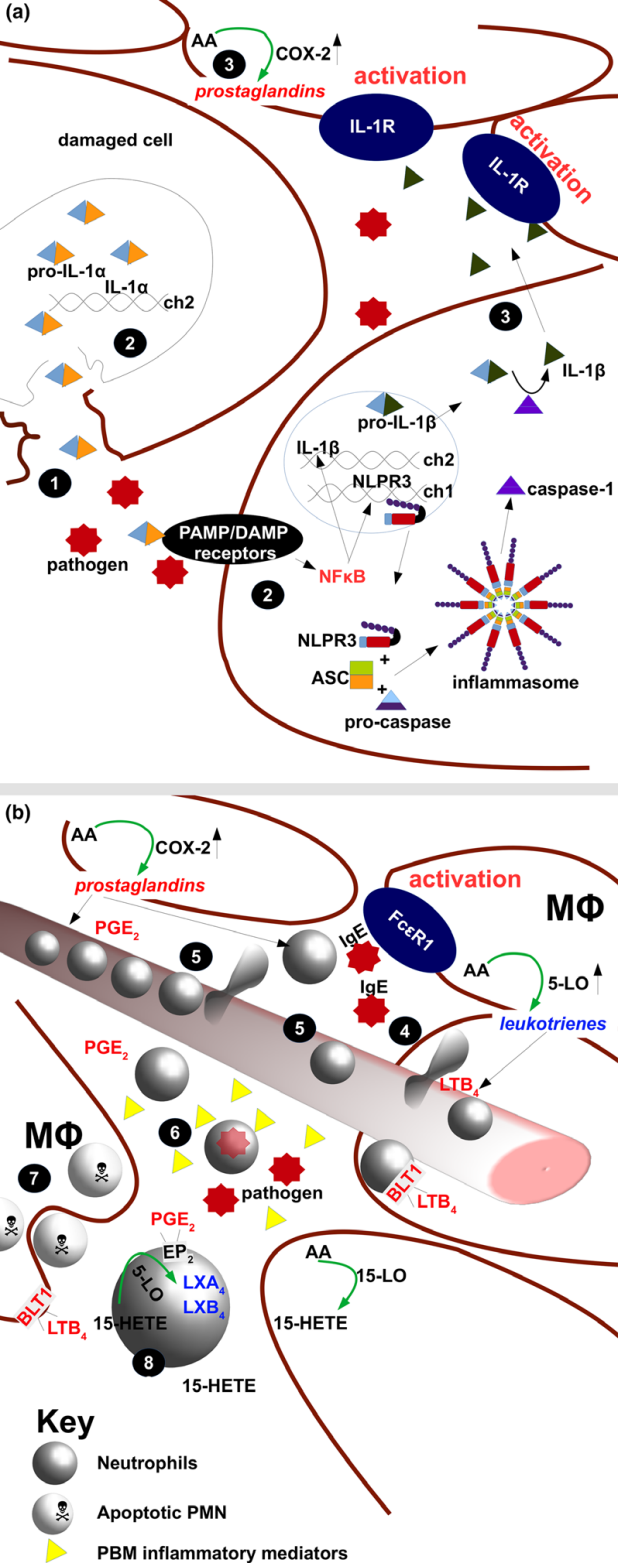
From initial damage to the establishment of inflammation. **(a)** 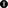 Cells damaged by injury release molecules, such as IL‐1α, delivering potent pro‐inflammatory signals via 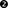 danger‐associated molecular pattern (DAMP) receptors, whilst pathogens are recognised by pathogen‐associated molecular pattern (PAMP) receptors. 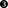 Signalling via both of these classes of receptors activate multiple additional processes directly or via the inflammasome, including changes in gene expression, secretion of bioactive molecules such as IL‐1β and PGE_2_. **(b)** The action of secreted cytokine and prostaglandins 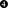 recruits innate immune cells to the area of damage and 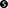
alters the behaviour of local endothelium initiating inflammation. 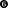 Innate immune cells, predominantly neutrophils, arrive and move from the blood vessels to the tissue and phagocytose or kill pathogens, and then die. 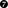 Other innate cells, such as macrophages, phagocytose dead and dying neutrophils contributing to a reduction in pro‐inflammatory signalling. 15‐LO, 15‐lipoxygenase; 5‐LO, 5‐lipoxygenase; AA, arachidonic acid; ASC, apoptosis‐associated speck‐like protein; BLT1, leukotriene receptor; ch1, ch2, chromosome 1 or 2; COX‐2, cyclooxygenase 2; DAMP, danger‐associated molecule pattern; FcεR1, FC epsilon receptor 1; IgE, Immunoglobulin E; IL‐1R, interleukin 1 receptor; LTB_4_, leukotriene B4; Mϕ, macrophage; NLPR3, cryopyrin PAMP detector; PAMP, pathogen‐associated molecular pattern; PGE_2_, prostaglandin E2.

Early after injury, damaged cells release DAMPs such as IL (Interleukin)‐1α (Figure 1) whilst other pro‐inflammatory molecules are released by components of the initial inflammatory response, such as platelets, which accumulate to stabilise blood loss at the site of the damage. Other pro‐inflammatory signals are generated by stromal cells in response to changes in the homeostatic environment such as changes in oxygen tension^4^ and redox state of the tissue^5^ and alterations in the tissue extracellular matrix (ECM) which trigger signalling involving crosstalk of Wilms tumor‐1 (WT1), Yes‐associated protein (YAP)‐1 and E‐cadherin^6^ triggering epithelial–mesenchymal transitions (EMTs) as part of the wound repair process.^7^

The detection of PAMP/DAMPs induces intracellular signalling pathways that converge on the activation of NFκB and the subsequent increased transcription of multiple genes, including pro‐IL‐1β, tumor necrosis factor α (TNFα) and the NLRP3 component of the inflammasome. Increased inflammasome activity drives the activation of pro‐IL‐1 β production, and release of this and other cytokines results in a strong pro‐inflammatory response. Stromal cells respond by secretion of additional pro‐inflammatory cytokines such as IL‐6, the level of which is critical in driving fibrosis (reviewed by Garth *et al*.^8^). This early response includes the release of growth factors, chemokines, nucleotides (ATP or ADP), prostaglandins and leukotrienes, from both stromal cells and platelets,^9^, ^10^, ^11^ the induction of new patterns of gene expression and proliferation, the activation of local endothelium, increased perfusion into the tissue and the recruitment of leukocytes. Importantly, IL‐1 signalling strongly influences the upregulation of cyclooxygenase‐2 (COX‐2) activity leading to enhanced biosynthesis of a range of prostaglandins, which contribute to the inflammatory cascade.^12^

Leukocytes which express a range of PRR are rapidly recruited to the damaged tissue and become activated by PRR‐driven recognition of pathogens that they encounter. The modulation of the local vasculature, the initiation of platelet binding proximal to the tissue damage and the activation of endothelium adjacent to the damage lead to an influx of neutrophils and other leukocytes, local oedema and an increase in the supply of nutrients to the damaged site. Neutrophils which dominate the influx express multiple Toll‐like receptors (TLR) and are specifically activated by ligands associated with encountered pathogens or released from damaged tissue cells that contain conserved structural elements. The importance of the rapidity of this response mediated first via recruitment of granulocytes from the bloodstream is indicated by neutropenic cancer patients experiencing a much higher relative risk of mortality.^13^

If the host has pre‐existing humoral immunity to the invading pathogens, interactions occur with the innate immune response. Low‐affinity type I Fcγ receptors (FcγR), expressed heterogeneously on leukocytes,^14^ do not bind monomeric IgG subtypes, but are activated by pathogen‐specific immunocomplexes. Circulating anti‐pathogen immunoglobulins bind their specific antigen, and the resulting immunocomplexes are bound by Fc receptors leading to protective immune responses. Such binding induces the activation of effector leukocytes,^15^ and cross‐linking of FcγR’s on granulocytes triggers degranulation and antibacterial activity,^16^ with the cross‐linking of FcγRIIIa expressed on NK cells similarly resulting in cellular activation and degranulation of effector molecules.^17^ Additionally, certain parasite antigens are recognised by specific IgE antibodies typically bound to FcεRI expressed on tissue‐resident mast cells and basophils triggering immediate‐type hypersensitivity reactions.^18^

In the absence of pre‐existing immunity, the activation of neutrophils and other leukocytes such as tissue‐resident immune cells such as alveolar macrophages,^19^ dendritic cells (DC^20^) and tissue memory resident lymphocytes (TMR^21^) is regulated by the concentration of the PAMPs or DAMPs present in the damaged tissue. Activation of neutrophils results in their degranulation of toxic molecules, such as proteases and the active or passive release of neutrophil extracellular traps (NETs^22^) that destroy pathogens but also cause damage to host tissue, adding to the level of DAMPs and further activating additional tissue cells. This results in a further amplification of the inflammatory response via the recruitment and the secretion of pro‐inflammatory cytokines by tissue‐resident cells.^23^

In particular, secretion of IL‐12 and IL‐23 by local DC influences the differentiation T helper (Th)‐1 cells and Th17 cells, respectively.^24^, ^25^ The stimulation of Th17 cells by IL‐23 potently induces IL‐17^26^ and IL‐22 secretion from Th17 cells recruited to the damaged tissue.^27^ The production of IL‐22 by bone marrow‐derived DC in response to stimulation by selective PAMPs further suggests that tissue‐resident DC may also be an additional contributor to IL‐22 levels.^28^ Both IL‐17 and IL‐22 stimulate lung epithelial cells resulting in the secretion of a broad range of pro‐inflammatory chemokines. Additionally, IL‐22 elicits the release of a range of antibacterial peptides from lung epithelial cells, which synergise to enhance anti‐pathogen responses.^27^ As a counter to this positive feedback loop of pro‐inflammatory signalling, PAMP‐activated alveolar macrophages secrete IL‐27^29^ which has potent anti‐inflammatory effects including the blocking of IL‐17 secretion by Th17 cells, and the induction of immunomodulating receptors on effector T cells and T regulatory cells, leading to secretion of the anti‐inflammatory IL‐10.^30^ Additionally, activated iTr35Tregs (iTr35) secrete the related cytokine IL‐35 which has potent anti‐inflammatory properties mediated via the inhibition of T‐cell proliferation.^31^

## Maintenance of tissue structural integration supports regeneration

In parallel with the local immune response, tissue cells are stimulated to secrete extracellular matrix components and the proteases needed to remodel it. Cells proliferate and/or differentiate, and finally, excess cells undergo apoptosis in response to a reduction in the local levels of growth factors,^32^ resulting in the reforming of the previous tissue architecture. Tissue regeneration is dependent on a supply of cells from adjacent healthy tissue or precursor cell populations. The likelihood that such cells are available is related in part to the level of damage, natural history and possibly the age of the individual.^33^ If the stimuli which initiated the healing microenvironment are not reduced, cellular responses continue and this sustains the activation and differentiation of myofibroblasts.^34^ Consequently, repair processes dominate, and the damaged tissue is replaced mostly with fibrous scar tissue.

## Regeneration following acute but limited damage

In the lungs, the epithelial lining of the respiratory tract is highly susceptible to damage from airborne infection, irritants or airborne toxins. These linings readily regenerate following superficial damage which has not destroyed underlying tissue structure and extracellular matrix. In the lung, acute damage to the epithelium can stimulate an effective regenerative response driven by proliferative responses from healthy adjacent epithelium.^35^, ^36^

The importance that tissue remodelling plays during an effective regenerative process is shown by a study by Schiller and colleagues.^37^ This study measured the dynamics of extracellular components from the time of bleomycin‐induced lung damage, transient fibrotic repair at 2 weeks, to lung regeneration after 4–8 weeks. Bleomycin is a bacterial‐derived molecule whose toxicity relates to a selective inhibition of nucleic acid synthesis and which causes the death of range of epithelial cell types including AEC1, AEC II, Club cells and endothelial cells in the first 7 days following exposure. As a result of bleomycin‐associated cell damage, a large number of changes occur in the remaining tissue, and in particular, certain extracellular matrix proteins like Emilin2, which interacts with elastin, become highly upregulated.

During effective lung regeneration, the basement membrane underlying the epithelial and endothelial cell layers is thought to be critical in supporting stem cell migration into the damaged area through its provision of a scaffold^38^ and capacity to concentrate paracrine secreted bioactive molecules.^39^

At day 3, which corresponds to the peak of the inflammatory response following bleomycin treatment, there is a significant upregulation of secreted neutrophil elastase inhibitor, α2‐macroglobulin and a range of serine protease inhibitors (serpins),^37^ necessary to control the extent of protease‐mediated damage to structures such as the basement membrane. Similarly, there is evidence of enhanced expression of the proteoglycan molecules decorin and biglycan. These molecules bind to collagen I and can sequester secreted TGF‐β reducing it's signalling activity and the level fibrogenesis.^37^, ^40^ The presence of decorin and biglycan can affect the binding of fibroblasts, which initiates intracellular signalling for pathways associated with migration such as Rac1 and RhoA, and thus assists cellular migration.^41^ A sparsity of decorin because of reduced production by fibroblasts is associated with the chronic inflammatory environment associated with severe emphysema.^42^ *In vitro* studies have shown that decorin mRNA can be increased by the corticosteroid, dexamethasone (Dex), whilst biglycan mRNA is increased by all trans‐retinoic acid treatment (ATRA).^43^

## Lung repair following disruption of tissue structure

Under conditions of chronic inflammation, the continued recruitment and activation and degranulation of neutrophils lead to tissue damage mediated by proteases including elastase, which digests extracellular matrix proteins and can disrupt structure provided in the lung by the basement membrane that underlies the alveolar epithelium. When the structural integrity of the lung is damaged, repair processes dominate over regeneration. This is illustrated in an elegant study using the elastase chronic lung damage model where the amount of damage to the lung was titrated over 4 sequential weekly low doses of pancreatic porcine elastase (PPE).^44^ Following one instillation of elastase, there was not a significant reduction in alveolar septa elastin content but there was a significant infiltrate of mononuclear cells into the lung parenchyma. A second installation resulted in structural deformation of the alveoli and a reduced alveolar septa elastin content. However, it was not until a third instillation of elastase, which further decreased alveolar septa elastin content, that evidence of fibrosis was detected, as indicated by increased staining for collagen fibre content in the alveoli and small airways.

Recent comparisons of the similarities and differences of the damaging effects of silica, bleomycin and paraquat in an animal model suggest that these agents damage the lung in different and characteristic ways.^45^ Using a mouse model, pharyngeal aspiration of these agents all initiated an inflammatory response which peaked at day 7 post‐treatment. Although histopathology and genetic analysis demonstrated many similar characteristics between the 3 damaging molecules, there are clear differences in terms of nature of the histopathology, degree of immune involvement, level and type of cellular damage, level of ECM modification associated genes and interestingly different sets of chemokines.^45^ The greater number of unique immune‐associated genes activated following silica exposure may have important correlates for silica exposure in humans, where some patients have been reported with signs of autoimmune disease as well as fibrosis.^46^, ^47^ Whilst it appears that there are common drivers of the early proliferative and inflammatory responses occurring following lung damage, the distinct genetic responses identified following different types of damage suggest that damage‐related therapeutic targets should be investigated.

## Chronic lung disease results from dysregulated repair processes

The consequences of prolonged or repeated periods of infection, irritants or toxins can induce a state of chronic inflammation, a tissue environment in which repair processes that replace the normal tissue architecture with fibrotic tissue are favored over tissue regeneration. If multiple areas of the lung are affected in this way, then lung function is progressively and irreversibly reduced.

The lung’s structural characteristics result in dynamic and distinct environmental and mechanical properties which vary along the ventilatory tree from trachea to terminal alveoli. Insights into the differential susceptibility of lung structures have been gained via the integration of physiological measurements of intra‐airway pressure^48^ and ultrastructural analysis provided initially by stereology^49^, ^50^ and more recently through micro‐computerised tomography (μCT) studies.^51^ Such studies have contributed to the development of models which predict that the penetration of particles into the lung is related to their size and the air flow rate. A range of experimental and mathematical models highlight that particle deposition is not evenly distributed and the site of peak disposition shifts from distal lung regions to proximal regions with a surface dose that is much higher in conducting airways than within the alveoli.^52^, ^53^ Given that airway resistance and airway number increase with each level of branching and the narrowing of airway diameter, Hogg *et al*. proposed that small airways are susceptible to particle‐driven damage which precedes the collapse of terminal bronchioles, leading to life‐threatening increases in airway resistance, characteristic of the pathological progression of chronic obstructive pulmonary disease (COPD).^54^ Evidence of markers of cellular senescence in the lung cell populations of COPD patients is consistent with an overwhelming of the reparative capacity of the lung in response to ongoing stress and injury.^55^

In contrast, lung diseases such as pulmonary fibrosis do not seem to be predominantly driven by particle damage and subsequent chronic inflammation and are insensitive to anti‐inflammatory treatment.^56^ Rather, they are characterised by a heterogeneous pattern of progressive lung structural change, mediated by altered regulation or function of lung fibroblasts. Foci of proliferating fibroblasts are characteristic of IPF and are thought to reflect alveolar epithelial cell damage and subsequent collapse of the distal airspace.^57^ Whilst a range of exposures, including smoking, are considered independent risk factors for IPF, the causative agent/s have not been identified. In a recent study, the observation that all IPF patients examined showed an overexpression of the glycoprotein mucin 5b^58^ has prompted the consideration that aberrant mucociliary clearance might be promoting an altered lung microbiome^59^ generating damage because of innate immune responses that support IPF development. The detection of increased pulmonary expression in a small cohort of IPS patients of IL25, which is pro‐fibrotic in animal models and released by local populations of ILC2 cells, is consistent with this hypothesis.^60^

## Therapeutic approaches to modulate damage/enhance regeneration over repair

Current and proposed therapeutic approaches to halt chronic lung damage involve reducing lung inflammation, with the most common treatment regimens seeking a broad abatement of pro‐inflammatory signalling through the use of inhaled corticosteroids. However, as discussed above, current anti‐inflammatory approaches are not effective in treating IPF. An illustration on how corticosteroid treatment might have untended negative effects on damaged lung is provided in a recent study assessing the ability of Dex treatment to improve outcome following bleomycin‐induced lung damage. This study reported that Dex‐treated mice showed a reduction in tumor necrosis factor (TNF)‐α concentration in lavage fluid; however, there was no significant reduction in neutrophil numbers in Dex‐treated mice. Additionally, cultures of primary alveolar epithelial cells treated with Dex showed a delayed and dose‐dependent lower repair rate than saline‐control‐treated cultures.^61^

There are important clinical issues with the efficacy of these treatment regimens in humans including the development of steroid‐resistant inflammation^62^ which is associated with allergic asthma exacerbations involving neutrophil accumulation. The development of resistance to anti‐inflammatory corticosteroids is thought to be multifactorial including the high levels of pro‐inflammatory provided by increased expression of the pro‐inflammatory cytokine macrophage migration inhibitory factor (MIF), an excessive activation of the mitogen‐activated protein (MAP) kinase pathways which overwhelms the downregulating effect of the glucocorticoid receptor of the cyclic‐AMP response element binding protein (CBP) and the impaired nuclear recruitment of histone deacetylase‐2 (HDAC2), which again would normally act to downregulate the expression of pro‐inflammatory genes.^63^, ^64^, ^65^

## Targeting the inflammatory cascade to treat lung disease

The identification of DAMP and PAMP as molecules critical to the initiation and maintaining of inflammation has prompted pre‐clinical studies to assess the effectiveness of DAMP/PAMP blockade in the resolving inflammation. Whilst not technically a DAMP, IL‐1β is an important part of the signalling amplification that follows DAMP or PAMP activation of the inflammasome complex. There are three types of reagents targeting IL‐1 that have been evaluated for anti‐inflammatory activity in the context of lung diseases: anakinra is an IL‐1 receptor antagonist,^66^ MEDI8968 is a humanised anti‐IL‐1R antibody, and canakinumab is an anti‐IL‐1β blocking antibody. Whilst these approaches have been used successfully in murine models to reduce IL‐1‐associated inflammation and despite canakinumab and anakinra showing efficacy in the treatment of the rare inflammatory disease adult‐onset Still’s disease,^67^ no improved lung function measures have been detected in COPD clinical trials patients utilising canakinumab^68^ or MEDI8968.^69^ A recently completed clinical trial assessing the efficacy of canakinumab in the treatment of pulmonary sarcoidosis has not yet reported,^70^ whilst there is a proposed study to assess the efficacy of anakinra in the treatment of cystic fibrosis.^71^

The cytokine IL‐5 is a current anti‐inflammatory therapeutic target and has been detected in bronchial lung biopsies of asthmatics^72^ in which antigen‐specific CD4 T cells are responsible for its secretion.^73^ As IL‐5 can mobilise eosinophils from a bone marrow pool,^74^ its regulation is considered critical. Experimental evidence for the central role of IL‐5 is provided by the protection of mice which lack the IL‐5 gene from antigen‐driven models of allergic airway disease.^75^ These promising studies have prompted clinical trials looking to neutralise IL‐5 activity in the context of acute airway disease. A recent meta‐analysis covering the use of the anti‐IL‐5 treatment, mepolizumab, has concluded that when properly targeted to treat patients with severe eosinophilic asthma, that treatment both significantly reduced the total numbers of exacerbations per patient, and also the number of exacerbations requiring hospitalisation.

Other reagents being assessed for the treatment of lung disease include tocilizumab, a humanised monoclonal antibody which blocks the activity of the interleukin‐6 receptor (IL‐6R). This reagent has been trialled in the context of the interstitial lung disease (ILD) that is associated with systemic sclerosis in patients diagnosed for less than 2 years.^76^ Patients treated with tocilizumab showed preserved lung function relative to placebo‐treated patients. In a small off‐label study treating patients with progressive disease and who had failed other immunotherapy treatments, 4 of 9 showed a stabilisation of their disease.^77^

Numerous animal studies have demonstrated the role that the IL‐17 family of cytokines play in inflammatory lung disease and that blocking IL‐17a^78^ or IL‐17e also known as IL‐25^79^ reduces lung pathology. Additionally, inhibitors of phosphoinositide 3‐kinases (PI3K’s) which are activated following IL‐25 signalling have been shown to be effective in reducing asthma like airway hypersensitivity and airway inflammation.^80^ A humanised anti‐IL‐17a antibody, secukinumab, which has successfully been used to treat psoriasis, has been trialled in a cohort of corticosteroid‐resistant asthmatics without success.^81^

On balance, the failure to demonstrate improved outcomes following treatments with biologics directed against a range of inflammatory cytokines is discouraging but given the efficacy of such approaches in treating other inflammatory conditions, then additional dosing approaches or perhaps multi‐target biologics^82^ may lead to effective treatments in human lung disease.

**Figure 2:**
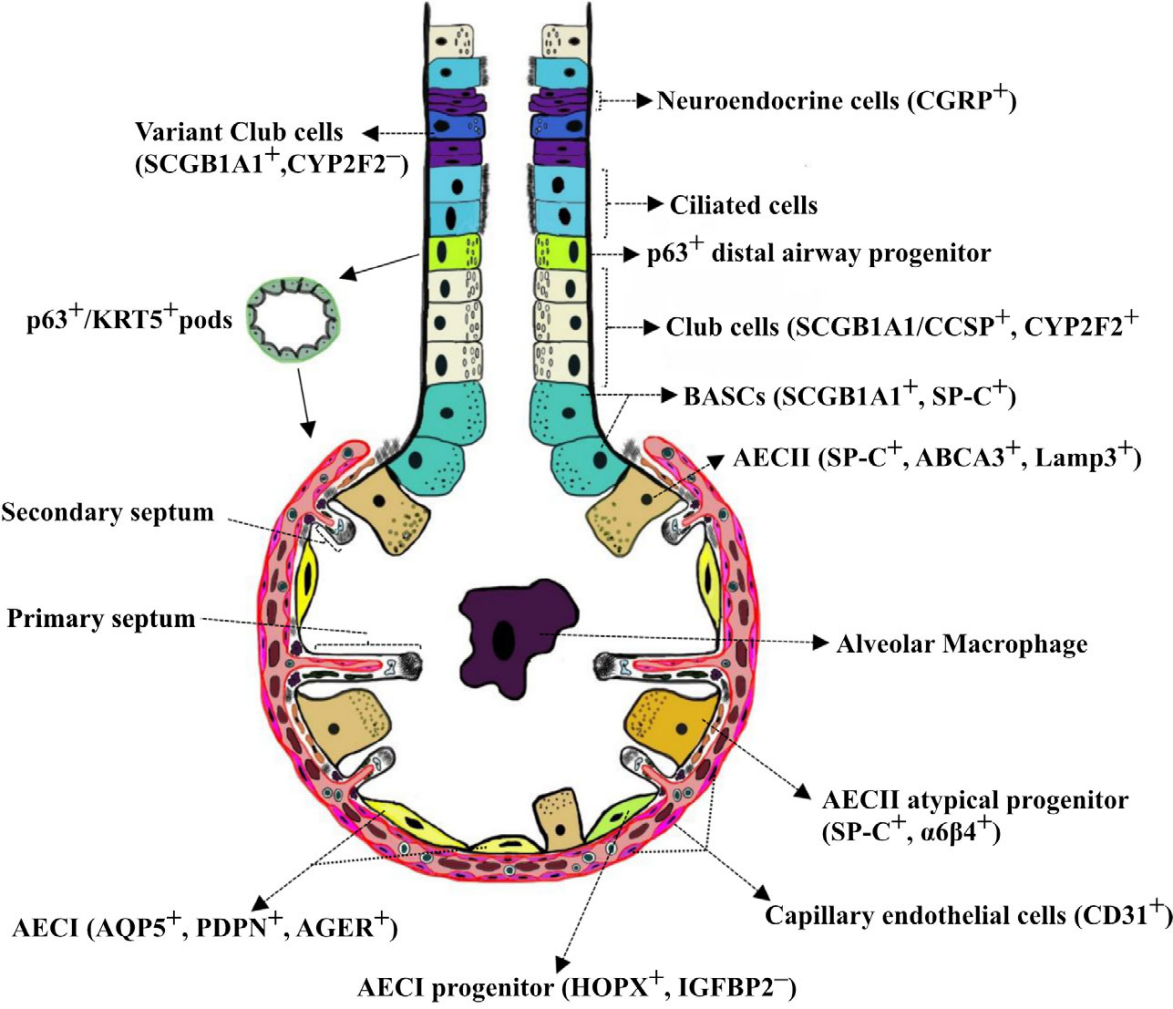
Localisation of epithelial cell progenitors that contribute to neoalveolarisation. Lineage‐tracing studies have highlighted a novel role for airway progenitor cells in alveolar repair and regeneration.^38^ CCSP^+^ club cells can self‐renew and give rise to ciliated cells to propagate airway repair. Club cells characterised by expression of CYP2F2 can differentiate into AECIIs and AECI cells. Club cells with a low expression of CYP2F2 (variant club cells located close to neuroendocrine bodies) are resistant to naphthalene‐induced injury and help regenerate denuded airways and alveolar regions. Activation of p63^+^ progenitors following H1N1 influenza virus infection gives rise to p63^+^/KRT5^+^ pods that can migrate from the distal airway epithelium to denuded alveolar regions to participate in neoalveolarisation. BASCs located at the bronchioalveolar duct junction can differentiate to airway club cells and AECIIs. SP‐C^+^AECII surfactant‐producing cells are regarded as the classic progenitor for AECIs; however, a variant of AECIIs characterised by SP‐C^–^/α6β4^+^ expression constitutes an additional source of AECIIs and AECIs. AECIs, which cover 90% of the alveolar epithelium, are regarded as terminally differentiated and characterised by expression of AQP5, PDPN, HOPX and AGER. The plasticity of AEC1s has been demonstrated using lineage‐tracing techniques that revealed HOPX^+^/Igfbp2^–^AECIs can differentiate to AECIIs.^38^

As described above, the lung has the capacity to regenerate, especially the lung epithelium, a process that is dependent on the survival of suitable progenitor cells located within a viable distance of the damage site. Recent fate lineage tracking of alveolar progenitor cells in mice describes an ongoing but infrequent generation of new alveolar units, with ATII cells acting as progenitors for ATI cells^38^ (Figure 2). Following specific damage to ATI by hyperoxygenation, large numbers of ATII cells contribute to renewal of the ATI population. Importantly, the authors detected an intact ATII compartment in both young and aged mice.^33^ The phenotype of additional progenitor cells has been defined by various surface markers including bronchioalveolar stem cells (BASC) at the bronchioalveolar ductal junctions, multipotent epithelial stem/progenitor cells^83^ and lineage‐negative progenitor cells, which proliferate following injury. The contribution of mesenchymal stromal cells to RLG has not been elucidated, although McQualter has shown evidence that the proliferation of epithelial stem/progenitor cells is dependent on the growth factors released by resident mesenchymal stromal cells in the adult lung.^83^ A recent study has demonstrated that mesenchymal stromal cells from older mice proliferate poorly in response to pneumonectomy compared to young mice.^33^ Moreover, new alveolarisation requires new septa formation driven by alpha‐smooth muscle actin^+^ interstitial myofibroblasts that are derived from a sub‐population of platelet‐derived growth factor‐alpha^+^ fibroblasts that respond to peroxisome proliferator‐activated receptor‐γ (PPAR‐γ).^84^ In addition, there is evidence that bone marrow‐derived cells contribute to RLG.^85^ Immune cells such as alveolar macrophages are potential sources of growth factors and proteases likely to be critical to the tissue remodelling. Thus, it is likely that precursor, resident and infiltrating cells are all necessary for successful lung regeneration and defects in any of these populations might limit regenerative potential.

The demonstration of the pluripotency of mesenchymal stem cells (MSC) has naturally suggested that they might play apart in regenerative processes. The adoptive transplantation of MSC has been shown in multiple studies to have pro‐regenerative and anti‐inflammatory effects, often when they accumulate in lung tissues. For example, adoptively transferred MSC have shown positive benefits in the context of bleomycin‐induced lung injury in mice,^86^ an effect attributed to the production of IL‐1 receptor antagonist by the MSC. A common feature described in many MSC adoptive transfer protocols is the low frequency and longevity of MSC that are detected longer than 1 day following transfer. Further *in vitro* studies have demonstrated that exosomes, which are small membrane vesicles containing mixed concentrations of cytoplasmic molecules, are released from cultured adipose‐derived MSC. These exosomes both contain and express alpha‐1‐anti‐trypsin on their surface,^87^ a molecule which potently inhibits protease activity. These exosomes have been demonstrated to show anti‐elastase activity and provide a proof of concept for cell‐free delivery systems that might be used to down modulate proteases active at sites of chronic inflammation.

Other studies have looked at the effectiveness of treating lung damage with all trans‐retinoic acid (ATRA) given that it has a structural relationship to vitamin A and has been shown to be involved in lung development.^88^ A number of studies have published improved outcomes using ATRA following lung damage induced by elastase, in rats and mice.^89^, ^90^ Recently, Takeda *et al*.^91^ have demonstrated a potential amplification of the effect the restorative adoptive transfer of MSC into elastase damage mouse lung by simultaneously systemically treating with ATRA. They described that the MCS exerted a synergistic restorative response which included a reduction in the mean linear intercept distance, an increase in the compliance of the lung and an increase in the surface area of the lung. The authors provided evidence that the mammalian target for rapamycin (mTor) function, linked to the activity of the S6k1 cofactor kinase, was critical for the restorative effect and that the synergism with ATRA was blocked when mTor function was inhibited by the drug rapamycin.

**Figure 3:**
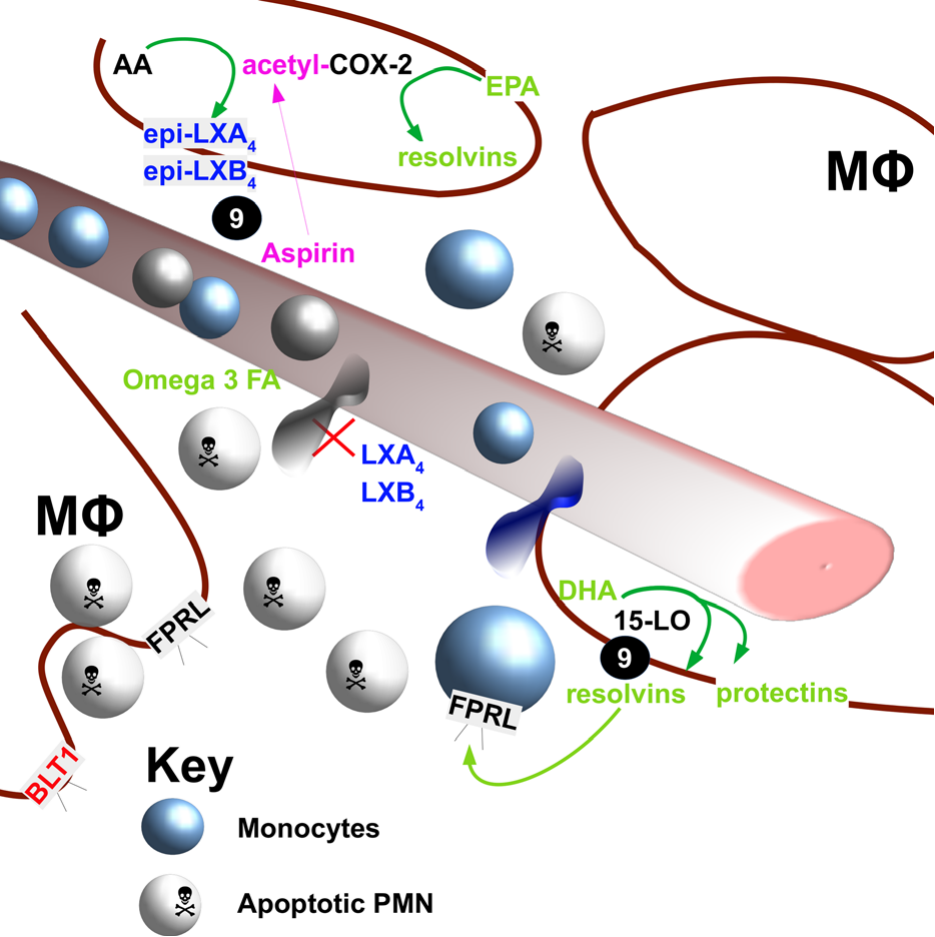
Repurposing of prostaglandin biosynthesis to generate wound‐resolving factors. 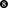 Subtle but profound changes in the metabolism of various fatty acid precursors are due to a change in the activity of enzymes such as lipoxygenases, and 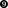 release anti‐inflammatory molecules such as lipoxins and resolvins, can change the balance from a pro‐inflammatory to pro‐resolving healing environment. 15‐HETE, 15‐hydroxyeicosatetraenoic; 15‐LO, 15‐lipoxygenase; 5‐LO, 5‐lipoxygenase; AA, arachidonic acid; BLT1, leukotriene receptor; COX‐2, cyclooxygenase 2; DHA, docosahexaenoic acid; EPA, eicosapentaenoic acid; FPRL, formyl peptide receptor like‐1; LXA_4_, lipoxin A4; LXB_4_, lipoxin B4; Mϕ, macrophage; Omega 3 FA, Omega 3 containing fatty acids; PGE_2_, prostaglandin E2.

As discussed earlier, the pro‐inflammatory mediators released by PAMP‐ and DAMP‐activated cells include prostaglandins which act on endothelial cells increasing local blood flow, on local nerves inducing pain and influence the function of leukocytes, including neutrophils. The cyclooxygenase (COX)‐1 and 2 enzymes are involved in the biosynthesis of prostaglandins and are the targets of the non‐steroidal anti‐inflammatory inhibitors, which include aspirin. Activation of neutrophils by the prostaglandin‐E_2_ (PGE_2)_ induces a switch from the production of the pro‐inflammatory leukotriene beta‐4 (LTB_4_) to the molecule lipoxin which acts to reduce further neutrophil recruitment (Figure 3). The production of other anti‐inflammatory molecules has also been discovered as a consequence of the study of the modulation of fatty acid biosynthesis during inflammation (reviewed by Serhan *et al*.^92^), including D and E‐series resolvins, protectins and maresins. These molecules are generated as either direct biochemical products of the polyunsaturated fatty acid biosynthetic pathways or in response to blocking of cyclooxygenase activity with molecules such as aspirin. The feedstock for these molecules is omega‐3 fatty acids, and this provides a mechanistic explanation for the associated health benefits of diets that contain foods rich in these lipids.^93^ The potential for the production of these molecules to be modulated *in vivo* or used therapeutically is just being established, with evidence that treatment with resolvin E1 could reduce lung inflammation in an apoptosis‐dependent manner,^94^ whilst studies that investigate administering so called immunonutrition are demonstrating some clinical efficacy in measurably increasing plasma resolvin E1 levels and decreasing plasma IL‐6 levels.^95^

These pre‐clinical studies give hope that even in badly damaged lungs, function can be partially restored, whilst it is clear that a deeper understanding of the mechanisms underlying lung restoration is still to be achieved.

## Conclusions

The challenge to prospectively assist in the recovery from lung damage and improve clinical outcomes lies in the developing of treatments that enhance regenerative over repair processes. As discussed above, the management of the inflammatory response appears key to these improvements. The application of anti‐cytokine agents now available in the clinic such as anti‐IL‐1 and anti‐IL‐6 might be considered then to modulate acute inflammatory responses, with potential benefits balanced against other clinical risk factors. For example, the enhanced risk of infection might be reduced by co‐treating with anti‐pathogen medications. Further understanding the scope and power of endogenous anti‐inflammatory molecules, such as resolvin‐like molecules, whose efficacy might be affected by the diet, has the potential to positively affect lung healing potential, at a population level.

## Conflict of interest

The authors declare no conflict of interest.
